# Characterisation of Alloy Composition of Protohistoric Small Boat Models from Sardinia (Italy)

**DOI:** 10.3390/ma15041324

**Published:** 2022-02-11

**Authors:** Roberta Iannaccone, Anna Depalmas, Claudio Bulla, Sergio Augusto Barcellos Lins, Antonio Brunetti

**Affiliations:** 1 Dipartimento di Chimica e Farmacia, Università degli Studi di Sassari, 07100 Sassari, Italy; riannaccone@uniss.it; 2 Dipartimento di Scienze Umanistiche e Sociali, Università degli Studi di Sassari, 07100 Sassari, Italy; depalmas@uniss.it (A.D.); cbulla@uniss.it (C.B.); 3 Dipartimento di Scienze di Base e Applicate per L’ingegneria, Università degli Studi di Roma “La Sapienza”, 00161 Rome, Italy; 4 Dipartimento di Scienze Biomediche, Università degli Studi di Sassari, 07100 Sassari, Italy; brunetti@uniss.it

**Keywords:** navicelle, Sardinian protohistoric bronzes, EDXRF, Monte Carlo simulation, Early Iron Age

## Abstract

The Sardinian protohistoric civilisation (Final Bronze Age and the Early Iron Age) has greatly contributed to the development of metallurgy in the Mediterranean area by producing a large number of bronze artefacts. Among them, small boat models (so-called “navicelle”) represent one of the most characteristic objects from the Sardinian Protohistoric civilisation. This work is an attempt to classify these boats on the basis of their alloy composition, provenience, and morphology. Due to the impossibility of removing the boats from the museum, alloys were characterised using energy-dispersive X-ray fluorescence spectrometry and Monte Carlo simulations. Fifteen boats were analyzed. Obtained results were compared to other performed analyses in the last few decades with different techniques and reported in the literature. Analyses allow for characterising both the bulk composition of ternary alloy Cu–Sn–Pb and patina thickness, offering useful information about their conservation status and the technological achievements of Sardinian craftsmen, while also providing information on smelting temperatures and casting techniques.

## 1. Introduction

The extraordinary development of metallurgy in Sardinia from the 14th century to the 10th century BCE was partially due to the presence of mineral deposits on the island, such as copper, silver, lead, and iron. Mining exploitation in the prehistoric and protohistoric periods is difficult to quantify, but references of the use of such mineral deposits were found [1,2,3].

In the Late Prehistoric period, the first contacts between Sardinia and other areas of the Mediterranean date back to the Middle and Late Bronze Age, as attested by the numerous examples of Mycenaean and Aegean artefacts found in settlements on the island, and to a lesser extent, by Sardinian exports to Crete and Cyprus. During the Final Bronze Age, there was a significant increase in extraneous goods to local production that appear to have been widely spread throughout the island as a result of the intensification of traffic passing through Sardinia and the evident direct involvement of the island [4].

Several authors emphasised the special relationship established with Cyprus, which acted as an agent in the transmission of objects, models, and, above all, technological know-how for metallurgical production, including the lost-wax casting technique in particular.

The evolution of metallurgy, referred to in much of the literature as ’Nuragic’, although it relates to a period after the construction of the nuraghi, which reached its maturity, complexity, and originality in the Early Iron Age, would seem to be distinctly marked by a process featuring a good capacity for imitation and the processing of allogenous products [5].

Evidence of the use of bronze alloy is attested since the beginning of the Bronze Age, reaching its peak at the end of the Final Bronze Age and the Early Iron Age [6].

Archaeological bronze replicas of boats (navicelle) represent one of the most characteristic bronze artefacts produced in the Early Iron Age (10th–middle 7th century BCE) in Sardinia. The navicelle represent an exceptional expression within the figurative production of the protohistoric period on the island, and show the high technical expertise reached by Sardinian workshops [7].

The production of these artefacts is associated with sacred sites, and with multiple meanings related to the sea and its symbolic value for this civilisation. They were likely used as votive oil lamps. Every boat is generally characterised by a hull, a suspension ring supported by a deck or/and mast, raised sides, and an animal’s head called a protome. These features are used together with other characteristics as classification criteria. Navicelle production involved the use of techniques such as the lost-wax casting of subsequently assembled single pieces [8].

The first documentation of a navicella dates back to 1663. Within the collection of cardinal Flavio I Chigi in Rome, the description of an ancient bronze ship appears. They were first studied by Alberto Lamarmora, who published extensive descriptions of pieces found during his 1840s “Voyage en Sardaigne” [7].

Only 150 authentic Nuragic navicelle have been discovered in Sardinia so far, with a major problem arising from their chronological dating and collocation. Many other boats probably exist, but may have been stolen by illegal diggers and are thereby hidden in illicit private collections. Most of the pieces found on the island are out of archaeological context, with a lack of information on provenance, position, and correlation with other materials in the excavation. Many findings were random and occurred during agricultural works, and sometimes acquisition was derived from recoveries of questionable origins; a few rare limited findings were discovered in closed unviolated areas [7,9].

We attempted to chronologically date navicelle on the basis of artefacts found in Etruscan and peninsular (Campania and Calabria) contexts, which constitute an essential temporal reference for materials exported from Sardinia. Despite their importance, only a few studies were carried out with noninvasive protocols to shed light on material provenance, manufacturing technology, and authenticity [6,8,9,10,11].

This work presents the application of a systematic noninvasive analytical methodology to characterise the materials of these peculiar artefacts through the analysis of a wide collection. The aim is to improve the knowledge of their production and provenance, combining energy-dispersive X-ray spectrometry (ED-XRF) data with Monte Carlo simulations to provide compositional data.

## 2. Materials and Methods

**Figure 1:**
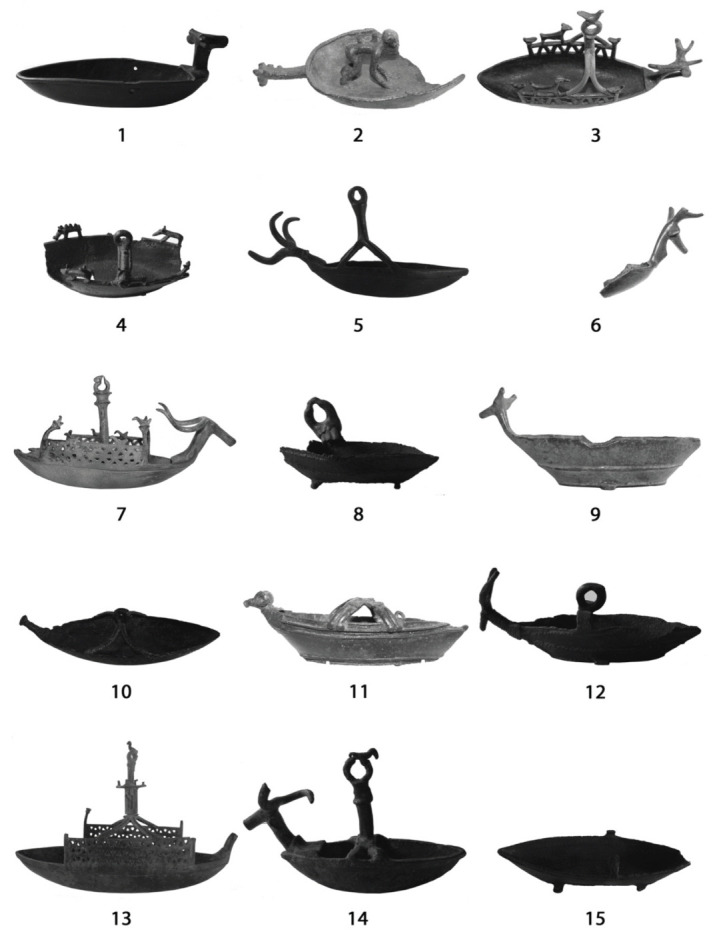
Images of analysed navicelle. (**1**). Ardara, Scala de Boes; (**2**). Baunei, Golgo; (**3**). Bultei, Bonotta; (**4**). Meana, Madaresu; (**5**). Mores, Monte Lecchesinus; (**6**). Ogliastra region, unknown location; (**7**). Orroli, Pipitzu; (**8**). Padria, San Giuseppe; (**9**). Posada, unknown location; (**10**). Tula, Badu ’e Trovu; (**11**). Tula, Badu ‘e Trovu; (**12**). Unknown location (n. inv. 1347); (**13**). Padria, Badde Rupida; (**14**). Uri, Su Igante; (**15**). Oliena, unknown location.

**Figure 2:**
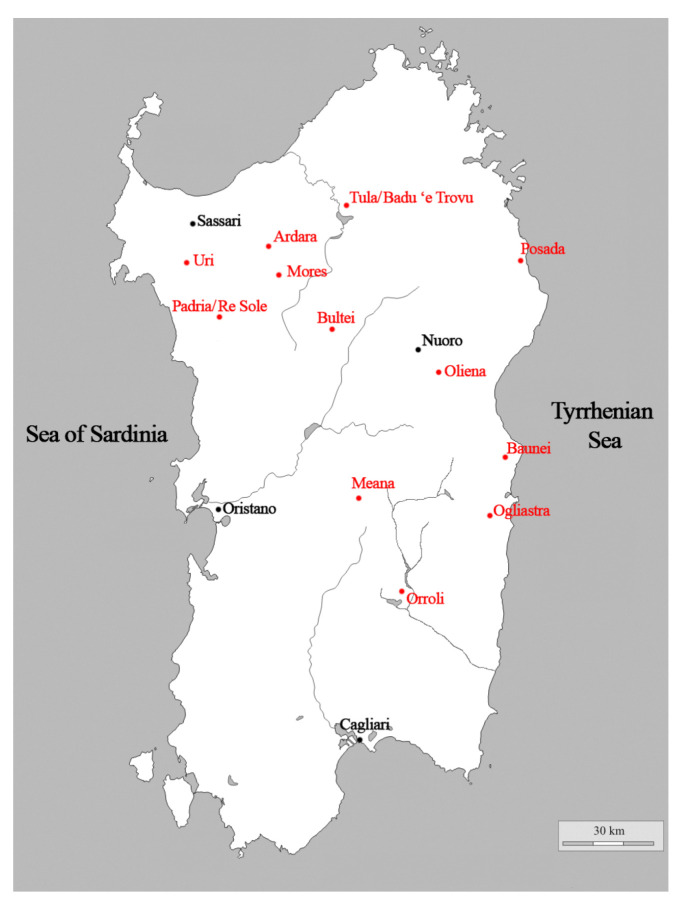
Map of Sardinia with indication (or estimation) of sites where the navicelle were found.

In total, 15 navicelle were analysed during the campaign: 6 belonging to the National Archaeological Museum of Cagliari and 9 belonging to National Archaeological Museum G.A. Sanna of Sassari. The boats were selected according to their importance and typological representativeness or their archeological context, attempting to select pieces from as many different sites as possible. This resulted in a set of selected navicelle coming from different protohistorical sites throughout Sardinia except for two from unknown locations (Figure 1 and Figure 2). The boats were classified according to the shape of the hull [7]; for our analyses, we took into account one sample for each type or variation.

EDXRF is a well-known nondestructive technique based on the interaction of X-rays with an examined sample. The outcomes of this interaction are expressed in a spectrum containing energy-based classification of the number of detected photons. The spectrum is formed by a background (continuum) with a series of fluorescence peaks superimposed to it. The central energy channel of each peak can be connected to tabled fluorescence lines, making it possible to identify chemical elements. The intensities of the peaks are roughly connected to the concentration of the corresponding element, while the background cannot be attributed to a specific chemical element, as it is created by a scattering event. However, it contains precious information regarding so-called obscure materials, i.e., materials of which the fluorescence cannot be detected due to their low fluorescence emission energy.

To extract information from the background and reproduce a complex structure of the surface of the analysed sample, the experiment and its respective outputs were simulated with a Monte Carlo code. The latter is based on a probabilistic algorithm that finds application in research areas where the high dimensionality of the problem forbids an analytical approach. In this case, it simulates the interaction of X-ray photons with the sample matter. To perform such simulations, the experimental setup geometry, X-ray tube emission profile, and detector must also be modelled inside the code. As may be expected, the simulation of such complex events is slow, but some fast and specialised codes are available for the specific simulation of EDXRF [12,13,14,15,16]. In this context, we used for the first time the XRMC code together with XMI-SIMS modules. Both codes are based on a constantly updated atomic parameter database named Xraylib [17]. They were tested and validated with reference samples producing many publications [18,19].

The applied simulation protocol was the following: after modelling the experimental setup, detector response, and X-ray tube emission, a ”guess” composition profile and the structure of the sample were provided. The structure of a bronze sample can usually be modelled as multilayered with often a protective layer (such as Paraloid), a patina layer, and the bulk alloy. In a real sample, the layers are commonly heterogeneous and/or rough, but an approximation to a flat, smooth, and homogeneous layer does not significantly affect the estimation. This preparatory step is followed by the first simulation. The obtained simulated and measured spectra are compared, and if differences are noticed (in the initial steps, this estimation is just visual), the structure and composition are accordingly changed. The last two steps are repeated until the differences are negligible. The last iterations’ differences are estimated by a chi-squared test. When the simulated spectrum is a good reproduction of the real one, the simulated model and composition can be considered to be a good approximation of the real sample. However, an objection can be raised: can a high-dimensional problem have more than one solution? This reasonable question was addressed, and many different alternative compositions and structures were tested even if an excellent reproduction of the measured spectrum had been obtained. In our rather extensive experience with this kind of simulation, only in one case did we find a double solution for a low concentration element (≲1%) contained in all layers. When this happens, the most conservative solution is chosen.

EDXRF measurements were carried out by using a custom portable XRF system with adjustable geometry, which allows for optimization betweenthe measurement geometry and sample surface. The measurement system was based on an X-ray tube with an Ag target (Mini-X by AMPTEK®, Bedford, MA, United States) and an X-123 silicon-drift detector (SDD) also manufactured by AMPTEK®. The detector was normally placed to the sample surface with an X-ray source at 30${}^{\circ }$ relative to the detector itself. The distance between detector and X-ray tube from the sample surface was around 2–3 cm. The X-ray tube worked at 35 kV and from 5 to 20 μA. Each spectral acquisition took 4 min [9].

After acquisition, all spectra were compared with a Monte Carlo model on the basis of two or three multilayered structures. The analysed samples were modelled as a two-layer structure: a patina layer, mainly composed of corrosion products (such as copper-based compounds, copper salts, and other elements [20]), superimposed to the bronze alloy layer. In some points, an additional layer of organic protective material was found, possibly applied during a past restoration [9]. In this case, a three-layer model was adopted, adding the protective layer to the previously described structure.

Table 1 summarises the name of each analysed navicella derived from its corresponding archaeological context, the museum of origin, and the abbreviation used in the diagrams.

Obtained data from analyses were subdivided and discussed into two main groups, hull and protome or other decorative elements, because of the possibility of obtaining the object assembly of two or more pieces. The literature and recent analyses [6] suggest that articulated objects were crafted in two or more phases, given the difficulty to fill up the entire mold with the molten metal and the likeliness of a lack of soldering know-how from the craftsman of the time [21].

## 3. Results and Discussion

All analysed spots on the entire group of 15 boats showed a ternary alloy composed of copper, tin, and lead as major components, with the presence of minor elements such as iron, arsenic, antimony, and zinc (<2.0 wt %) that can be considered as natural impurities of the ore.

The spectra also showed the presence of calcium, probably due to the surface enrichment of soil elements, indicating the interaction between corrosion products and soil components [22].

This type of alloy composed of copper, tin, and lead was commonly used during the period spanning from the Late Bronze Age to the Classical and Hellenistic ages, mainly for casting small decorative objects [23].

**Figure 3:**
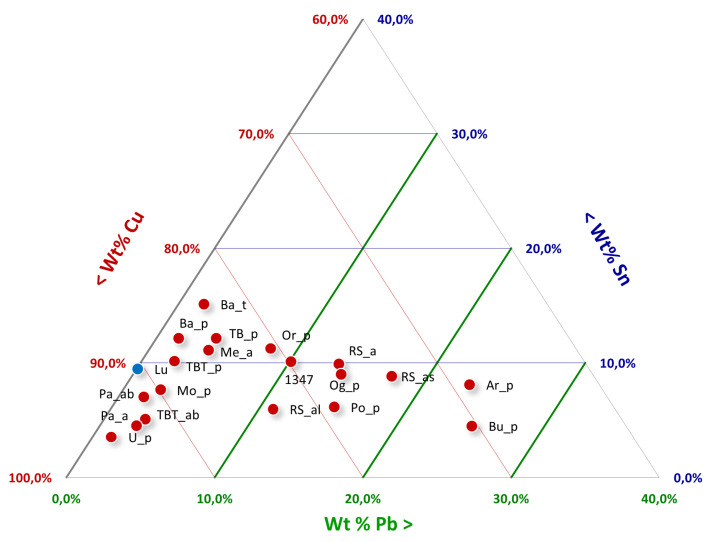
Ternary diagram for Cu–Sn–Pb with an indication (blue dot) of the boat composition model.

The composition of each navicella is reported in Table 2 and in the ternary diagram of Cu–Sn–Pb shown in Figure 3. Except for two navicelle (Ardara—Scala de Boes and Bultei), where the lead content was above 20 wt %, all other samples could be separated into groups. Figure 3 also shows in blue the Vetulonia boat composition obtained from TOF neutron diffraction analyses in 2019 [8].

In some cases, total weight percentage data showed a discrepancy with already published data [24] because the accuracy of the Monte Carlo method was improved and sensibility was revised.

**Figure 4:**
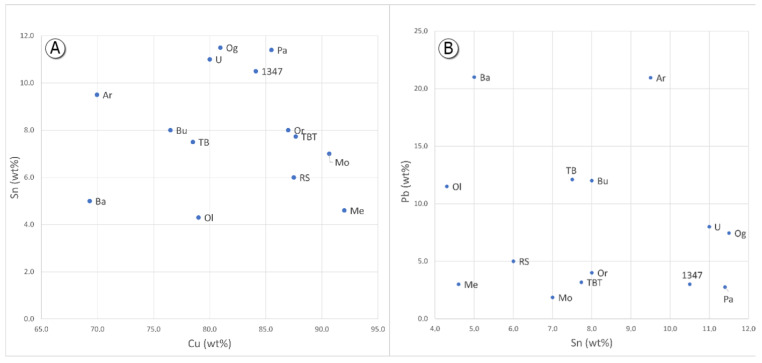
(**A**) Cu/Sn diagram, and (**B**) Sn/Pb diagram.

The average copper content obtained for the boats’ hulls spanned from 65 to 92 wt %, while tin, lead, and their respective ratios varied widely from one sample to another. Figure 4 shows the copper versus tin and copper versus lead plots for the composition of the hulls. There was no evidence for correlations based on the tin content, which would allow for a grouping of different measurements. In any case, all obtained results showed tin content higher than 4.0 wt %.

Tin content spanning from 4 to 12 wt % sorted all our samples into the region of low-tin bronze and under the standard limit of solid solution [6,22]. This content is comparable with the higher tin content detected in small decorative metalwork from Italic and Etruscan areas, reflecting the conservative tradition of Bronze Age alloying [25].

The increasing amount of tin, for a weight percentage in the range of 8%–10%, increases the alloy’s mechanical proprieties [22,26]. Contents exceeding 10 wt % show the problem of cracking due to the presence of the (α + δ) eutectoid phase. In our case, however, artefacts had mere decorative functions [27], and high mechanical properties were not strictly needed.

In contrast, the plot of tin content versus lead shows three different groups (Figure 4). The first group includes hulls where lead was under 5 wt %, the second group includes lead content between 5 and 15 wt %, and the last group comprises only two samples with lead percentages above 15 wt %.

**Figure 5:**
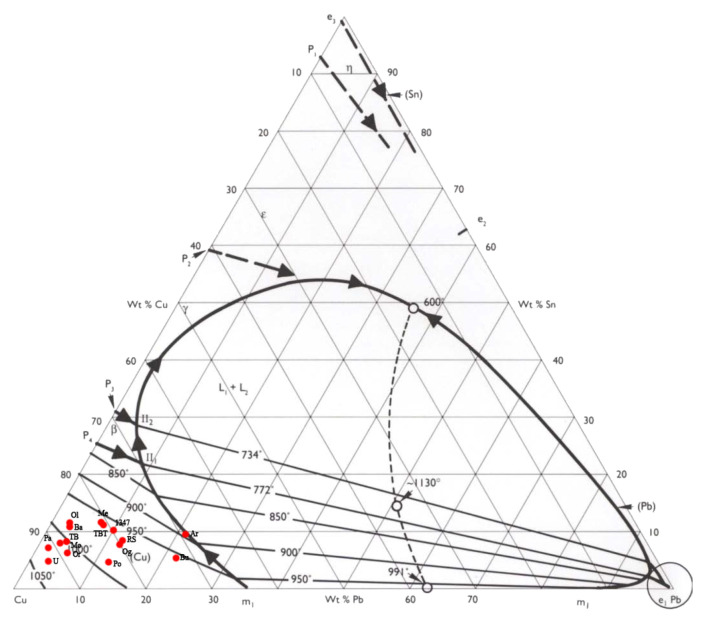
Ternary phase diagram of navicelle hulls.

The results of the alloy composition (for the major components) are reported in Figure 5. Samples fell in the miscible part of the ternary system with a liquidus temperature range from 900 to 1050 ${}^{\circ }$C. The sole exception is the Ardara sample, which fell in a region slightly above the 900 ${}^{\circ }$C line and close to the boundary between two liquid phases [23]. The fact that nearly all alloys fell into the miscible part of the copper–tin diagram may be an indicator of the use of a visual inspection to ascertain or control furnace temperature (e.g., verifying the colors of the flame and charcoal) as already suggested in the literature [27,28].

Generally, for ternary alloys with lead content below 30 wt %, the Cu–Sn solid solution starts to solidify, while lead remains in liquid form until the temperature falls under the eutectic point, creating a separate structure inside the alloy. The presence of lead decreases the melting point of the mixture compared to the binary Cu–Sn alloy, and increases the fluidity of the melt, easing the molten metal casting [23,29]. In the case of the Bultei boat, the articulated structure of the ship’s decoration suggests that the lead might have been intentionally added to obtain a more fluid melting.

Although this hypothesis may be realistic for the Bultei ship, the Ardara ship showed a very simple structure. The Bultei boat also showed the presence of iodine in the hull’s patina. This element, belonging to the stable halogens, is widely diffused on coastal areas soil, especially in combination with the presence of organic materials and clay, which helps retain it into the soil [30]. Other sources of iodine are marine plants such as seaweed, sedimentary rocks, and marine sediments [30].

Even if the origin of iodine is still not clear, the cuprous state becomes more stable as the covalent character of the Cu–X bond increases, usually with polarisable ligands or ligands capable of accepting electrons from copper, such as halogen. In addition, the very low solubility of such compounds could prevent leaching from the surface, preserving its presence up to the present day [31].

The thickness of the oxidation layer and its density vary from ship to ship, and can be an indication of the long-term corrosion of the alloy [32]. The average thickness for the hull’s patina is 90 μm, spanning from 20 μm, such as the one found in the Mores boat, to 300 μm, as that found in the Baunei ship. Further, the thickest layers presented considerably low density. The observed values fell into the average corrosion thickness of a great number of bronze samples belonging to the Bronze Age and Iron Age periods [33]. Moreover, most of the samples presented the same thickness value on both sides of the hull. Exceptions are the Padria and Oliena boats, whose thicknesses slightly differed between the two sides. In the Padria ship, the increase in thickness corresponds to an enrichment of copper in the corrosion layer, which in turn may correspond to the selective dissolution of copper [33].

**Figure 6:**
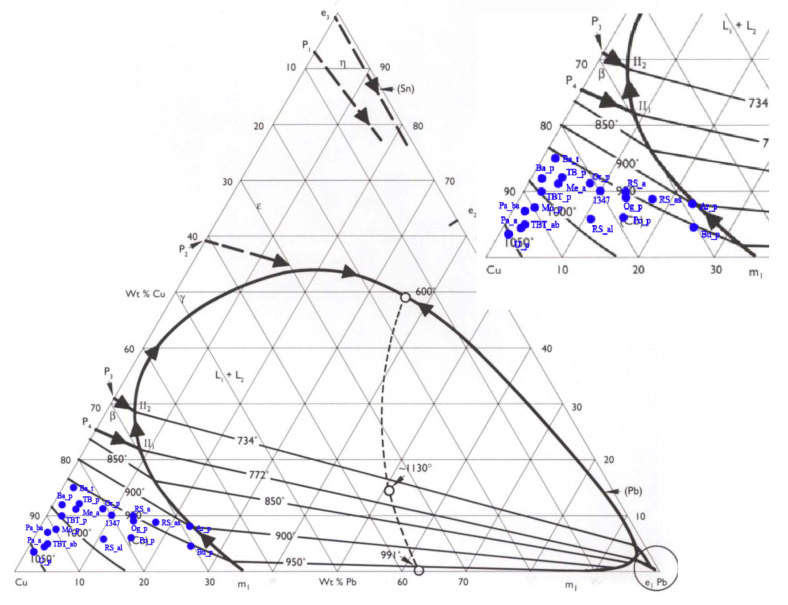
Ternary phase diagram and magnification of graphic areas with protome (XX_p) and other elements.

A separate discussion must be carried out for the protome and decoration elements, which can vary both in composition and conservation status when compared to the hulls. Figure 6 shows the ternary phase diagram for these decorative parts, where different compositions for some elements when compared to the hull were observed.

The EDXRF technique is essentially surface analysis, where the collected information is limited to circa 200–300 μm under the surface. Moreover, due to the corrosion and the different mobility of the chemical elements composing the alloy, there is an enrichment in lead and/or tin at the surface [34]. Despite these phenomena that can affect the estimation of the chemical composition of the alloy, only relative comparisons between chemical concentrations at different parts of the same boat are reported, so the conclusions about the relative concentrations remain valid.

Comparing the hull with the protome and other decorative elements, some ships showed a lower tin content in the hull (Baunei, Bultei, Orroli, Meana, Uri, Tula—Badde, Ogliastra, and Posada), which suggests that the melting temperature of the hull was higher than that of the other components.

In addition, comparing the tin content of decorative elements with the hull, some (mast, bridge, and ring) showed low percentages of tin. These differences in tin content can imply the use of the casting-on technique for additional parts [8] by arranging the fusion chamber directly on the hull for the protome (if present) or using a separate clay model for rings and other additional decorations, such as the mast [8].

This hypothesis is also supported by the comparisons among melting temperatures reported in the two ternary phase diagram discussed before.

In particular, the Re Sole boat hull was cast at a lower temperature than the lower segment of the mast was, while the upper part of the mast and the ring were probably cast at a temperature lower than 950 ${}^{\circ }$C.

A significant difference was observed between the protome of the Orroli boat and its hull. In this case, the protome was obtained by casting the molten alloy at a temperature close to 950 ${}^{\circ }$C, while the hull was possibly cast at a temperature up to 1000 ${}^{\circ }$C. This difference is also reflected in the amount of detectable tin in the bulk of both boats. These data can provide information on the casting procedure, suggesting the use of the casting-on procedure instead of a single casting of the entire artefact [8].

The two other ships (Uri and Tula, Badu ’e Trovu), showed the opposite behaviour. The protome was cast at a higher temperature than the hull was. In the Uri ship protome, the temperature was slightly higher, above 1050 ${}^{\circ }$C. A similar case was reported for the ship from Nuraghe Colovros in Lula’s territory [6], suggesting the casting of the additional part before the hull.

**Figure 7:**
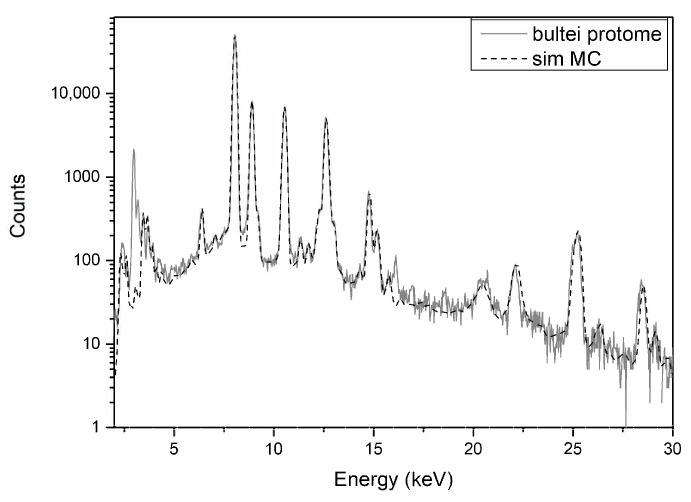
XRF spectra of protome from Bultei ship in grey, and MC simulation with in dotted line.

Another exception involves Bultei’s protome. It’s simulated and experimental spectra can be seen in Figure 7, where a lead content of 24.6 wt % is found, even higher than the already-high content found in the hull. This increase in lead content is not connected to a decrease in the two other main elements, copper and tin, or to the thickness of corrosion patina, which showed the highest value among all simulations.

High lead content was also detected in the Mores’ protome. In this case, the investigated point showed 25.5 wt % lead, while the hull’s average was 3.2 wt %. This element could have been intentionally added to obtain a more fluid casting metal [21]. On the other hand, the protome and the decorative elements of the Tula (Badu ’e Trovu) ship showed a decrease in lead content when compared with the hull in favour of an increase in copper content.

Copper values above 85%, such as those found in some parts of the boat models from Golgo, Orroli, Tula (MAN_CA and MAN_SS), Uri, Mores, Padria—San Giuseppe and Oliena were compared to the values recorded by Riederer 1980 [35] in the analyses carried out on the bronze boat models from public and private collections presented in the Karlsruhe exhibition of 1980, which showed wide variability in tin content, ranging from 3.65 to 12.60 and apparently not related to the copper value.

**Figure 8:**
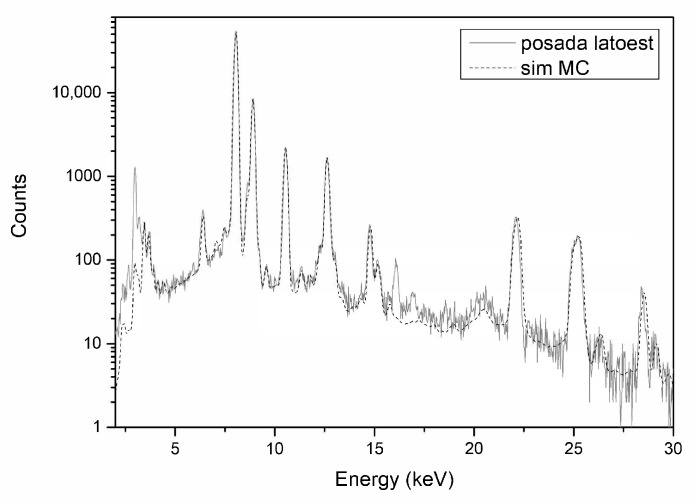
XRF spectra of Posada ship’s hull in grey, and MC simulation in dotted line.

A high lead content such as the one found in the boats from Posada (11.5/14.2), shown in Figure 8, Mores (*protome*: 25.5), Ogliastra (9.0/15.2), Padria-Re Sole (10.7/16.9), Bultei (21.0/24.6), Ardara (20.0/22.9) was not recorded in the boats analysed by Riederer.

Another interesting observation is the presence of silver in all three analysed spots in the Posada ship, above the threshold set to define it as a trace element. Nevertheless, it is still difficult to differentiate between an intentional addition or not. Copper objects with a discrete percentage of silver are attested in the Italian continent already during the Neolithic and Eneolithic periods [36]. Even if the metal was already known at least from the IV millennium BCE, the smelting was attested only in the Early Eneolithic [36]. The presence of this element could be related to the use of silver-enriched galena ore in the smelting phase or to an intentional addition. The presence of silver in the galena minerals could be observed, for example, in the Montevecchio-Ingurtosu district, in southwestern Sardinia, or Argentiera in the northwest [27,37].

Among the trace elements, iron, antimony, and zinc are interesting from a smelting technology perspective. Iron contents with an average of over 0.3 wt % can be considered to be a marker for a more efficient smelting process [38]. Antimony was reported in the Tula—Badu ’e Trovu and Posada boat alloy. Usually, antimony is proportional to the presence of arsenic, and both suggest the use of sulphude ore. In Tula–Badu ’e Trovu, arsenic content was lower than 0.1 wt % and under the detectable limit, while the Posada boat showed both elements.

## 4. Conclusions

EDXRF analyses refined with Monte Carlo simulations can provide useful information regarding the composition and conservation conditions of navicelle. The reported compositions in the ternary alloy diagram showed the extensive expertise of the metalworkers from ancient Sardinia. The use of a narrow temperature range for casting the metal pieces demonstrates a fine control over the furnace temperature, which may have been produced through visual inspection by assessing the flame and charcoal colors.

All analysed alloys belonged to the low-tin bronze category, generally used from the Late Bronze Age to the Hellenistic age, consisting of a ternary alloy (copper, tin, and lead) and small percentages of other elements such as iron, zinc, arsenic, antimony, and silver.

The patina found on the surface of the boats showed an average thickness of 90 μm, spanning from 20 μm in some few cases to 300 μm, and, most often presenting a low density, which can be a marker for long-term corrosion processes or burial periods.

The data also showed a possible technique used to cast the objects. Besides the lost-wax technique, the use of the casting-on technique is hypothesised with the second melting of protome and decorative parts on the hull.

Lastly, the adopted analytical protocol allowed for highlighting some special cases, such as the high lead content in the Bultei and Ardara hulls, and Mores’ protome, the presence of silver in the Posada ship, and the different techniques used to cast the different parts of the Uri and Tula—Badu ’e Trovu boat models.

## Figures and Tables

**Table 1:** Overview of analysed samples with assigned name, museum provenance, their used abbreviations, and their number in Figure 1.

Navicella Provenance	Catalogue and Table Number in Typology [7]	Collocation Museo Archeologico Nazionale (MAN)	Acronym	Figure 1
Ardara, Scala de Boes	39 (tab. 32)	MAN di Sassari	Ar	1
Baunei, Golgo	2 (tab. 2)	MAN di Cagliari	Ba	2
Bultei, Bonotta	95 (tab. 73)	MAN di Cagliari	Bu	2
Meana, Madaresu	38 (tab. 31)	MAN di Sassari	Me	4
Mores, Monte Lecchesinus	70 (tab. 55)	MAN di Sassari	Mo	5
Ogliastra, unknown location	82 (tab. 61)	MAN di Cagliari	Og	6
Orroli, Pipitzu	87 (tab. 65)	MAN di Cagliari	Or	7
Padria, San Giuseppe	15 (tab. 13)	MAN di Sassari	Pa	8
Posada, unknown location	23 (tab. 20)	MAN di Cagliari	Po	9
Tula, Badu ’e Trovu	34 (tab. 27)	MAN di Sassari	TBT	10
Tula, Badu ’e Trovu	25 (tab. 21)	MAN di Cagliari	BT	11
Unknown location (inv. n. 1347)	11 (tab. 12)	MAN di Sassari	1347	12
Padria, nuraghe Badde Rupida (Re Sole boat)	84 (tab. 62)	MAN di Sassari	RS	13
Uri, Su Igante	62 (tab. 49)	MAN di Sassari	U	14
Oliena, unknown location	17 (tab. 15)	MAN di Sassari	Ol	15

**Table 2:** Main elements are given in wt % (±5%). Dashes indicate absence or quantity <2 wt %.

Navicella	Copper (Cu)	Tin (Sn)	Lead (Pb)	Antimony (Sb)	Iron (Fe)
Ar hull	69.6	9.4	20.9	-	-
Ar protome	68.0	8.0	22.9	-	-
Ba hull	84.1	10.5	3.0	-	2.0
Ba monkey head	82.6	15.0	1.7	-	-
Ba protome	85.6	12.0	1.5	-	-
Bu hull	69.3	5.0	21.0	-	2.0
Bu protome	69.0	4.4	24.6	-	-
Me hull	81.0	11.5	7.5	-	-
Me mast	83.9	11.0	4.0	-	-
Mo hull	87.7	7.7	3.2	-	-
Mo protome	64.0	5.6	25.5	-	4.5
Og hull	78.5	7.5	12.1	-	-
Og protome	76.9	9.0	9.0	-	-
Ol hull	85.5	11.4	2.8	-	-
Or hull	87.5	6.0	5.0	-	-
Or protome	79.0	11.0	8.0	-	-
RS hull	76.5	8.0	12.0	-	2.0
RS mast small	71.0	8.5	16.9	-	2.0
RS mast large	81.0	5.8	10.7	-	-
RS ring	73.9	9.5	13.0	-	3
P hull	90.7	7.0	1.9	-	-
P mast	91.0	7.0	1.7	-	-
P ring	92.9	4.5	2.5	-	-
Po hull	79.0	4.3	11.5	-	-
Po protome	76.3	5.8	14.2	-	-
TBT hull	80.0	11.0	8.0	-	-
TBT mast	91.4	5.0	2.8	-	-
TBT protome	86.3	10.0	2.2	1.2	-
BT hull	87.0	8.0	4.0	-	-
BT protome	83.0	12.0	4.0	-	-
1347 hull	79.0	10.0	10.0	-	-
U hull	92.0	4.6	3.0	-	-
U protome	95.0	3.5	1.3	-	-

## Data Availability

The generated data are available upon request to the authors.
